# Restoring activity‐dependent synaptic and microglial transcriptomic alterations in late‐stage Alzheimer’s Disease

**DOI:** 10.1002/alz.095712

**Published:** 2025-01-09

**Authors:** Amira Latif‐Hernandez, Robert R. Butler III, Patricia Moran‐Losada, Tao Yang, Kevin C Tran, Harry Liu, Tony Wyss‐Coray, Frank M. Longo

**Affiliations:** ^1^ Stanford University, Stanford, CA USA; ^2^ Wu Tsai Neurosciences Institute, Stanford University, Stanford, CA USA

## Abstract

**Background:**

Activity‐dependent transcription is crucial in establishing the late phase of long‐term potentiation (L‐LTP), which is altered in Alzheimer’s disease (AD). TrkB and TrkC signaling‐activator (BD10‐2) reduces AD‐related synaptic dysfunction. We determined whether the synapse‐sparing effects of BD10‐2 are reflected in transcriptional changes following L‐LTP induction.

**Method:**

We orally administered BD10‐2 or vehicle to 13‐month old APP^L/S^ and WT mice, daily for 3 months. We measured L‐LTP‐dependent gene expression through RNA‐sequencing in hippocampal slices with or without induction of Theta‐Burst‐Stimulation (TBS)‐induced LTP.

**Result:**

L‐LTP induction failed in APP^L/S^‐Veh but was restored in APP^L/S^‐BD2‐10 mice similarly to WT‐veh. Analysis of overlap enrichment between mouse TBS‐dependent gene expression and human AD‐relevant modules, revealed: (1) greater downregulation of neuronal genes by TBS in WT‐Veh and APP^L/S^‐BD10‐2 than in APP^L/S^‐Veh; and (2) upregulation of microglial associated AD‐enriched genes by TBS in all groups.

**Conclusion:**

BD10‐2 prevented AD‐related LTP deficits and restored activity‐dependent AD‐related gene transcription.

